# The Role of SGLT1 and GLUT2 in Intestinal Glucose Transport and Sensing

**DOI:** 10.1371/journal.pone.0089977

**Published:** 2014-02-26

**Authors:** Pia V. Röder, Kerstin E. Geillinger, Tamara S. Zietek, Bernard Thorens, Hermann Koepsell, Hannelore Daniel

**Affiliations:** 1 ZIEL Research Center for Nutrition and Food Sciences, Biochemistry Unit, Technische Universität München, Freising, Bavaria, Germany; 2 Center for Integrative Genomics, Université de Lausanne, Lausanne, Switzerland; 3 Department of Molecular Plant Physiology and Biophysics, Julius-von-Sachs-Institute, Julius-Maximilians-Universität Würzburg, Würzburg, Bavaria, Germany; University of Barcelona, Spain

## Abstract

Intestinal glucose absorption is mediated by SGLT1 whereas GLUT2 is considered to provide basolateral exit. Recently, it was proposed that GLUT2 can be recruited into the apical membrane after a high luminal glucose bolus allowing bulk absorption of glucose by facilitated diffusion. Moreover, SGLT1 and GLUT2 are suggested to play an important role in intestinal glucose sensing and incretin secretion. In mice that lack either SGLT1 or GLUT2 we re-assessed the role of these transporters in intestinal glucose uptake after radiotracer glucose gavage and performed Western blot analysis for transporter abundance in apical membrane fractions in a comparative approach. Moreover, we examined the contribution of these transporters to glucose-induced changes in plasma GIP, GLP-1 and insulin levels.

In mice lacking SGLT1, tissue retention of tracer glucose was drastically reduced throughout the entire small intestine whereas GLUT2-deficient animals exhibited higher tracer contents in tissue samples than wild type animals. Deletion of SGLT1 resulted also in reduced blood glucose elevations and abolished GIP and GLP-1 secretion in response to glucose. In mice lacking GLUT2, glucose-induced insulin but not incretin secretion was impaired. Western blot analysis revealed unchanged protein levels of SGLT1 after glucose gavage. GLUT2 detected in apical membrane fractions mainly resulted from contamination with basolateral membranes but did not change in density after glucose administration.

SGLT1 is unequivocally the prime intestinal glucose transporter even at high luminal glucose concentrations. Moreover, SGLT1 mediates glucose-induced incretin secretion. Our studies do not provide evidence for GLUT2 playing any role in either apical glucose influx or incretin secretion.

## Introduction

The worldwide increase in obesity and diabetes mellitus type 2 [1] has brought new interest to the mechanisms by which dietary monosaccharides are absorbed in the intestine and to which extent these systems undergo diet-dependent regulation. Glucose derived either from hydrolysis of starch or from sucrose is taken up into the epithelial cell predominantly by the sodium-dependent glucose co-transporter SGLT1 [2]–[4]. Its pivotal role in glucose absorption is demonstrated by the inability of animals lacking SGLT1 to survive on glucose-containing diets. However, *sglt1* knockout mice maintained on a glucose−/galactose-free diet are viable, healthy and fertile although they exhibit impaired intestinal glucose absorption [5]. Efflux of glucose from enterocytes into blood is thought to be mediated by GLUT2 as a facilitative uniporter with a low affinity but high transport capacity [6].

Already in 1933, Wertheimer classified intestinal sugar transport as determined by two components – one inhibited by phlorizin (today known as the most potent SGLT1 inhibitor) and a phlorizin-insensitive part [7]. It was later demonstrated that the component inhibited by phlorizin was saturable and electrogenic in nature [8] and that it contributed to water absorption [9]. Whereas this phlorizin-sensitive system was identified as SGLT1 [10], the phlorizin-insensitive component remains unknown. Pappenheimer and Reiss proposed a paracellular pathway to act as solvent drag [11] but this was not generally accepted. Interest in this second system emerged again when *in vivo* perfusion studies in rats suggested that GLUT2 could be recruited from intracellular vesicles into the apical membrane allowing in turn bulk quantities of glucose to be absorbed at high luminal glucose loads [12]–[17]. However, trafficking of GLUT2 into the brush border membrane was shown to require SGLT1 [18]. The fact that the methods applied play a critical role in the ability to prove translocation of GLUT2– even in rats – becomes evident as for example in one study the presence of apical GLUT2 was demonstrated in perfused, isolated intestinal segments [13] whereas the same group failed to show this in everted intestinal sleeves [19]. To our knowledge, only one study using mice is available in which GLUT2 trafficking was demonstrated using an everted gut ring technique and a brush border membrane vesicle transport assay [20]. Although GLUT2 was detected by Western blot in brush border membrane preparations [5], [20]–[22] only one study simultaneously applied a basolateral marker protein for control of cross-contamination [20].

Besides their role in glucose transport, both, SGLT1 [5], [23] and GLUT2 [24], [25] are proposed to function as glucose sensors in enteroendocrine cells leading or contributing to glucose-induced secretion of the incretins glucose-dependent insulinotropic peptide (GIP) and glucagon-like-peptide 1 (GLP-1).

Recent studies in morbidly obese humans as well as in obese mice suggest that GLUT2 may be present permanently in the apical membrane of enterocytes, thereby contributing to overshooting postprandial glucose levels [26]. Hence, assessing the contribution of SGLT1 and GLUT2 to overall intestinal glucose transport is crucial to understand normal physiology but also in view of their role in the pathophysiology of obesity-related impairments of glucose homeostasis. With the availability of both, mice lacking either SGLT1 or GLUT2, we were able to study the contribution of both transporters to intestinal glucose transport and their role in incretin hormone secretion under identical experimental conditions. We used intragastric administration of D-glucose including radiolabeled glucose and determined tracer contents in intestinal tissues along the entire small intestine as well as in plasma. We also assessed changes in blood glucose levels as well as in incretin and insulin concentrations following glucose gavage.

## Methods

### Mice

*Sglt1* wild type (*sglt1^+/+^*; C57BL/6 background) and knockout (*sglt1^−/−^*; 129/OLA-C57BL/6 background [5]) animals as well as *glut2* wild type (*glut2^+/+^*, C57BL/6 background) and RIPGLUT1×GLUT2 knockout mice (in this work referred to as *glut2^−/−^*; 129/Sv-C57BL/6 background with a re-expression of GLUT1 in pancreatic *β*-cells [27]) were bred and kept in the animal facilities of the Research Center of Nutrition and Food Sciences (ZIEL). All animals had free access to water and were fed standard chow (V1534 R/M-H; ssniff Spezialdiäten GmbH, Germany) except for the *sglt1* knockout animals which received a sugar-free diet composed of 33.8% protein, 30.7% fiber and 20.5% fat as well as vitamins and minerals (Altromin Spezialfutter GmbH & Co. KG, Germany). One week prior to the experiments, *sglt1* wild type animals as well as *glut2* wild type and knockout mice were fed the same sugar-free diet to exclude any diet-specific effects on transporter protein densities and function. For all experiments animals were deprived of food at 8 a.m. for 6 hours.

### Ethics Statement

All experiments were strictly conducted according to the recommendations of the Federation of European Laboratory Animal Science Associations (FELASA) and approved by the District Government of Upper Bavaria – veterinary medicine and consumer protection (Regierung von Oberbayern – Veterinärmedizin und Verbraucherschutz; permit number: 55.2-1-54-2531-39-10).

### Measurement of Intestinal Glucose Concentrations

Wild type mice received a glucose bolus of 4 g/kg body weight. After 15 minutes, animals were killed by cervical dislocation. Luminal fluids were collected and glucose concentrations were determined using a Glucose Hexokinase FS* assay kit (DiaSys Diagnostic Systems GmbH, Germany).

### Oral Administration of Radiolabeled Glucose and Quantification in Intestinal Tissues and Plasma

Mice were challenged with a radiolabeled glucose solution using a feeding tube. Unlabeled glucose of 40% that was shown previously to be sufficient to activate GLUT2 trafficking into the apical membrane [20] was combined with 370 Bq/µl [^14^C(U)]-D-glucose (Hartmann Analytic GmbH, Germany) and additionally with 370 Bq/µl [1-^3^H(N)]-D-Mannitol (Hartmann Analytic GmbH, Germany), the latter one allowing to correct glucose uptake into tissue for the adherent extracellular fluid phase. The glucose bolus was administered to a final dose of 4 g/kg body weight which accounted for 160 mM ±23 mM glucose in luminal fluids. After 15 minutes, animals were anesthetized with isoflurane for blood collection from the retro-orbital venous plexus and subsequently killed by cervical dislocation. Blood was centrifuged at 1200×g for 20 minutes before ^14^C tracer contents in plasma were measured in a liquid scintillation counter (PerkinElmer Inc., USA). The whole small intestine was everted and washed thoroughly in ice cold Krebs buffer (119 mM NaCl, 4,7 mM KCl, 2,5 mM CaCl_2_, 1,2 mM Mg SO_4_, 1,2 mM KH_2_PO_4_, 25 mM NaHCO_3_, pH 7.4). Defined intestinal segments of 1 cm were produced by means of razor blades fixed in exact 1 cm intervals on a rod. Incorporated radioactivity was measured and then used to calculate the glucose retention expressed as nmol per cm of tissue over 15 minutes. For statistical comparisons the average amount of glucose per cm as the sum of all segments divided by the number of segments was calculated.

### Analysis of Incretin Hormones and Insulin in Plasma

GIP, GLP-1 and insulin levels were measured in plasma before and 15 minutes after mice were challenged with 4 g/kg glucose. Blood glucose was measured at the tail vain with an Abbott FreeStyle Lite glucometer (Abbott Laboratories, USA). Animals were killed with CO_2_ before blood from the *vena cava inferior* was collected in EDTA tubes (Sarstedt AG & Co, Germany) and immediately mixed with DPP-IV inhibitor (EMD Millipore Corporation, USA). Total GIP levels in the plasma were determined with a rat/mouse total GIP ELISA kit (EMD Millipore Corporation, USA). Active GLP-1 levels in the plasma were measured using a high sensitivity GLP-1 active chemiluminescent ELISA kit (EMD Millipore Corporation, USA). Plasma insulin levels were determined with an ultra-sensitive mouse insulin ELISA kit (Crystal Chem, Inc, USA).

### Brush Border Membrane Isolation and Western Blot Analysis

Brush border membranes (BBM) were isolated by MgCl_2_ precipitation [5] from animals before and after receiving a 4 g/kg glucose gavage. 15 minutes after the bolus, mice were killed, mucosa was scraped off and snap-frozen until BBM isolation. Frozen mucosa was homogenized in ice cold M100 buffer (100 mM mannitol, 2 mM HEPES/Tris, pH 7.4) containing protease inhibitors and PMSF. Homogenates were incubated for 15 minutes with MgCl_2_ (20 mM final concentration). After low-speed centrifugation (3000×g, 15 minutes), supernatants were centrifuged for 30 minutes at 36000×g. The resulting pellet was resuspended in ice cold M300 buffer (300 mM mannitol, 20 mM HEPES/Tris, pH 7.4) containing protease inhibitors and PMSF. MgCl_2_ incubation and following centrifugation steps were repeated and final pellets were re-suspended in M300 buffer and snap-frozen until use. Samples (30 µg/lane) from *sglt1* and *glut2* wild type and appropriate knockout animals were run on the same SDS-gel. Nitrocellulose membranes were incubated over night at 4°C with primary antibodies raised against SGLT1 (custom made, raised against amino acids 586–601, 1∶15000), GLUT2 (Santa Cruz, C-19; 1∶250), Actin (Santa Cruz, C-11; 1∶5000) and Na-K-ATPase (abcam EP1845Y; 1∶50000). The specificity of the SGLT1 antibody was confirmed by the absence of the ∼ 90 kDa band in *sglt1* knockout animals. Secondary antibodies (LI-COR Biosciences, USA) were fluorescent dye-conjugated donkey anti-goat (IRDye800CW; 1∶12000) and donkey anti-rabbit (IRDye680RD; 1∶12000). Protein was visualized and quantified using Odyssey Infrared Imaging systems and software (LI-COR Biosciences).

### Immunofluorescence

Paraffin-embedded jejunal sections of 6 µm thickness were unmasked in citrate buffer pH 6.0 for 5 minutes at 95°C, blocked in 1% BSA and incubated with primary antibodies (SGLT1∶1: 250; GLUT2∶1: 200; see BBM isolation) diluted in PBS-T (0.05% Tween-20) over night at 4°C. Incubation with secondary antibodies (DAPI: 1∶1000 for nuclei staining; Cy3-conjugated donkey anti-goat/donkey anti-rabbit: 1∶250) diluted in PBS-T was performed for 1 hour at room temperature. Localization of SGLT1 and GLUT2 was examined using confocal microscopy (FluoView FV10i-DOC, Olympus; 60× oil lens).

### Statistical Analyses

Results are expressed as mean ± SEM. For statistical analyses, values representing tracer contents in intestinal tissues are expressed as the average per 1 cm segment ± SEM, that is total tracer content of the entire small intestine divided by the length of the intestine in cm to correct for differences in intestinal length. Differences were evaluated by one-tailed unpaired t-test ± Welch’s correction and 2-way ANOVA with Bonferroni post-tests (GraphPad Prism 4.01).

## Results

### SGLT1-deficient Mice Exhibit Markedly Reduced Intestinal Glucose Absorption and Glucose Levels in the Blood

**Figure 1:**
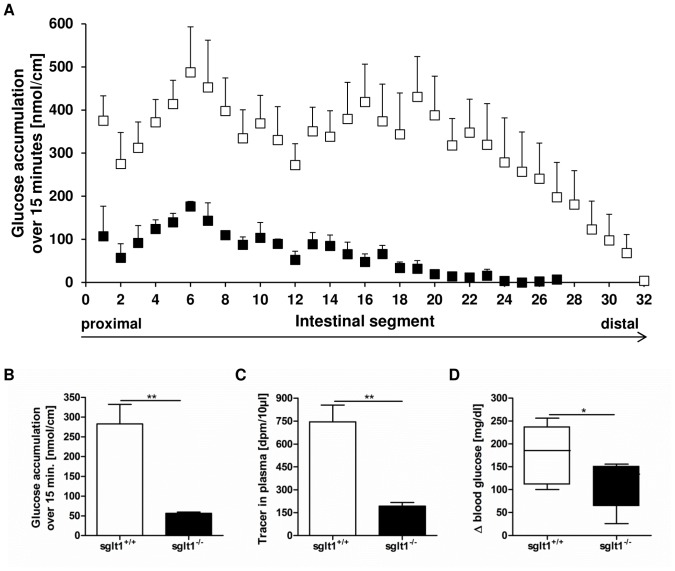
Deletion of SGLT1 results in reduced glucose contents in intestinal tissues and blood. *Sglt1* ^+/+^ (white bars) and *sglt1^−/−^* mice (black bars) received an intragastric glucose bolus (4 g/kg) containing radiolabeled D-glucose. After 15 minutes, radiotracer contents in intestinal tissue samples covering the entire small intestine, in plasma as well as blood glucose was measured. (A) Tissue profiling for glucose tracer contents along the small intestine of *sglt1* ^+/+^ and *sglt1^−/−^* mice. (B) Average accumulation of glucose tracer amounts in 1 cm intestinal tissue samples over 15 minutes. (C) Radiolabeled glucose contents in plasma. (D) Increase in blood glucose after glucose gavage. Values are expressed as mean ± SEM. Statistical analyses for glucose tracer in tissues and plasma were performed using unpaired t-test with Welch’s correction. ** *p*<0.01. Values of rise in blood glucose are expressed as mean ± SEM. Statistical analyses were performed using unpaired t-test. * *p*<0.05. N = 4–5 mice per group.

Animals lacking SGLT1 displayed significantly decreased glucose tracer contents in all segments of the intestine after the gavage when compared to wild type mice (Fig. 1A). The reduction accounted for 80% (Fig. 1B), confirming the prime role of SGLT1 in intestinal glucose absorption. The amount of radiotracer in plasma was reduced by 73% when compared to wild type animals (Fig. 1C) as was the rise in blood glucose levels (Fig. 1D).

### Loss of SGLT1 Results in Decreased Incretin and Insulin Responses to Glucose

**Figure 2:**
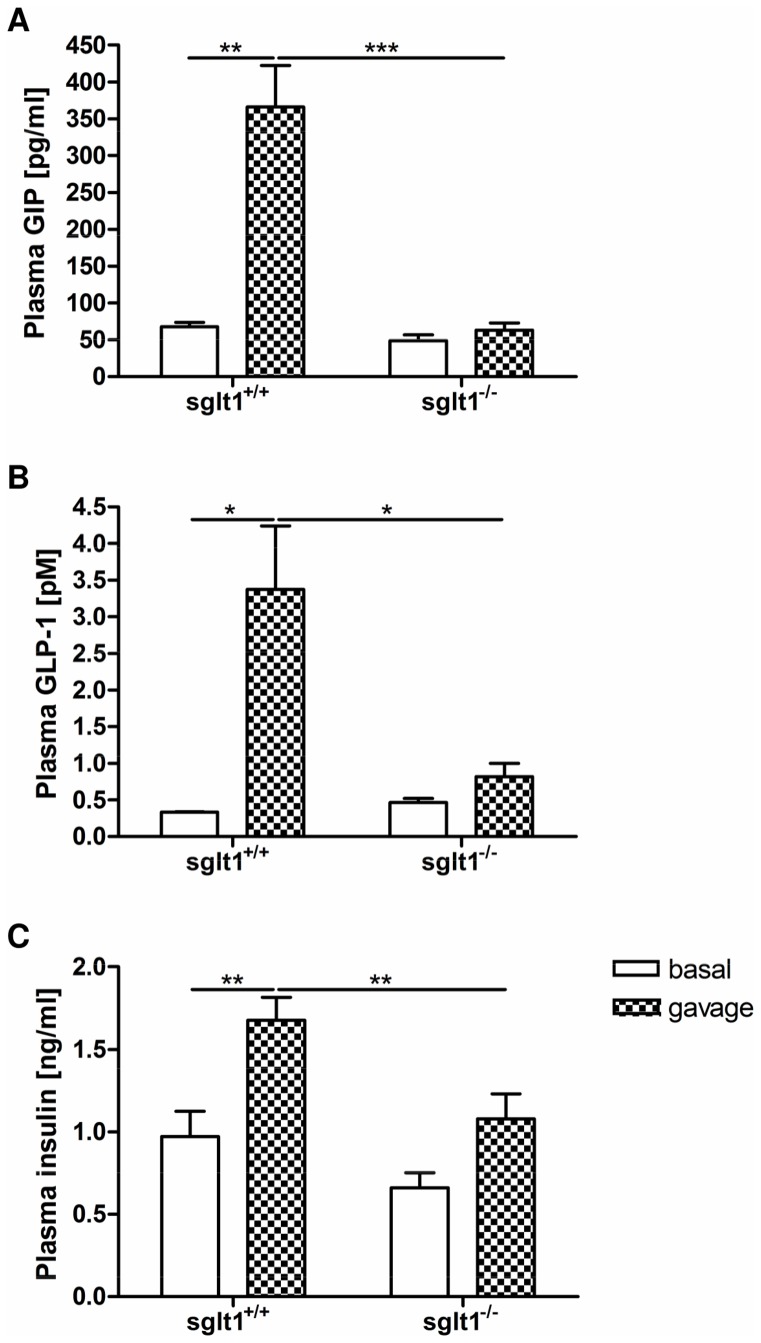
Increases in plasma GIP and GLP-1 levels after intragastric glucose are abolished in SGLT1-deficient mice. *Sglt1^+/+^* and *sglt1^−/−^* mice were challenged with an oral glucose gavage (4 g/kg). Plasma GIP, GLP-1 and insulin hormone levels were measured before (white bars) and 15 minutes after the bolus (plaid bars). (A) Total GIP concentrations in the fasting state (basal) and after glucose gavage. (B) Active GLP-1 concentrations in the fasting state and after glucose gavage. (C) Insulin concentrations in the fasting state and after gavage. Values are expressed as mean ± SEM. Statistical analyses were performed using 2-way ANOVA with Bonferroni post-test. * *p*<0.05, ** *p*<0.01, *** *p*<0.001. N = 3–8 mice per group.

After the glucose gavage, there was a more than 5-fold increase in plasma GIP levels in *sglt1^+/+^* mice when compared to basal levels; this response to glucose however, was completely abolished in *sglt1^−/−^* mice (Fig. 2A). Plasma GLP-1 concentrations increased around 10-fold after gavage in *sglt1^+/+^* but not in *sglt1^−/−^* animals (Fig. 2B). Plasma insulin levels following the glucose bolus were reduced by 36% in *sglt1^−/−^* as compared to *sglt1^+/+^* mice with a 1.7-fold increase as compared to basal levels (Fig. 2C). The abolition of GIP and GLP-1 responses in *sglt1^−/−^* mice demonstrate the pivotal role of SGLT1 also in intestinal incretin secretion. Despite the lack of incretin stimulation, insulin levels changed only modestly but this has also been observed previously [5].

### Mice Lacking GLUT2 Display Elevated Glucose Levels in Intestinal Tissues but Reduced Glucose Concentrations in the Blood After Oral Glucose Administration

**Figure 3:**
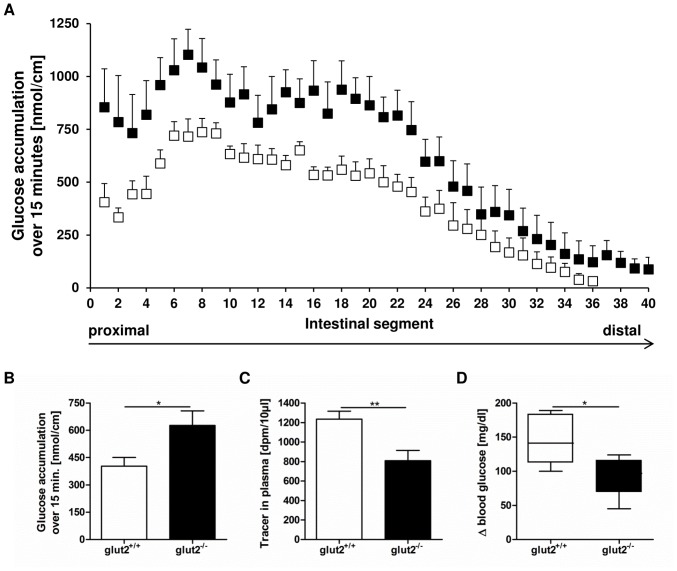
Animals lacking GLUT2 display higher tissue glucose contents but reduced amounts in blood. *Glut2^+/+^* (white bars) and *glut2^−/−^* mice (black bars) were administered an intragastric glucose bolus (4 g/kg) containing radiolabeled D-glucose. After 15 minutes, radiotracer content in intestinal tissue samples covering the whole small intestine, in plasma as well as blood glucose was measured. (A) Tissue radiotracer profile from proximal to distal small intestine in *glut2^+/+^* and *glut2^−/−^* animals. (B) Average accumulation of glucose tracer per 1 cm segment over 15 minutes. (C) Plasma tracer contents. (D) Increase in blood glucose levels after glucose load. Values are expressed as mean ± SEM. Statistical analyses were performed using unpaired t-test. * *p*<0.05, ** *p*<0.01. N = 5–6 mice per group.

15 minutes after the glucose bolus, *glut2* knockout mice exhibited significantly higher glucose tracer contents in intestinal tissues (Fig. 3A) exceeding those in wild type littermates by around 55% (Fig. 3B). The increased glucose retention in GLUT2-deficient animals suggests that the lack of GLUT2 impairs substrate efflux from intestinal cells into circulation which may explain the around 40% reduction in plasma radiotracer contents (Fig. 3C) and the significantly diminished elevation of blood glucose concentrations after the glucose load (Fig. 3D).

### GLUT2 Effects on Glucose-induced Incretin Secretion are Modest

**Figure 4:**
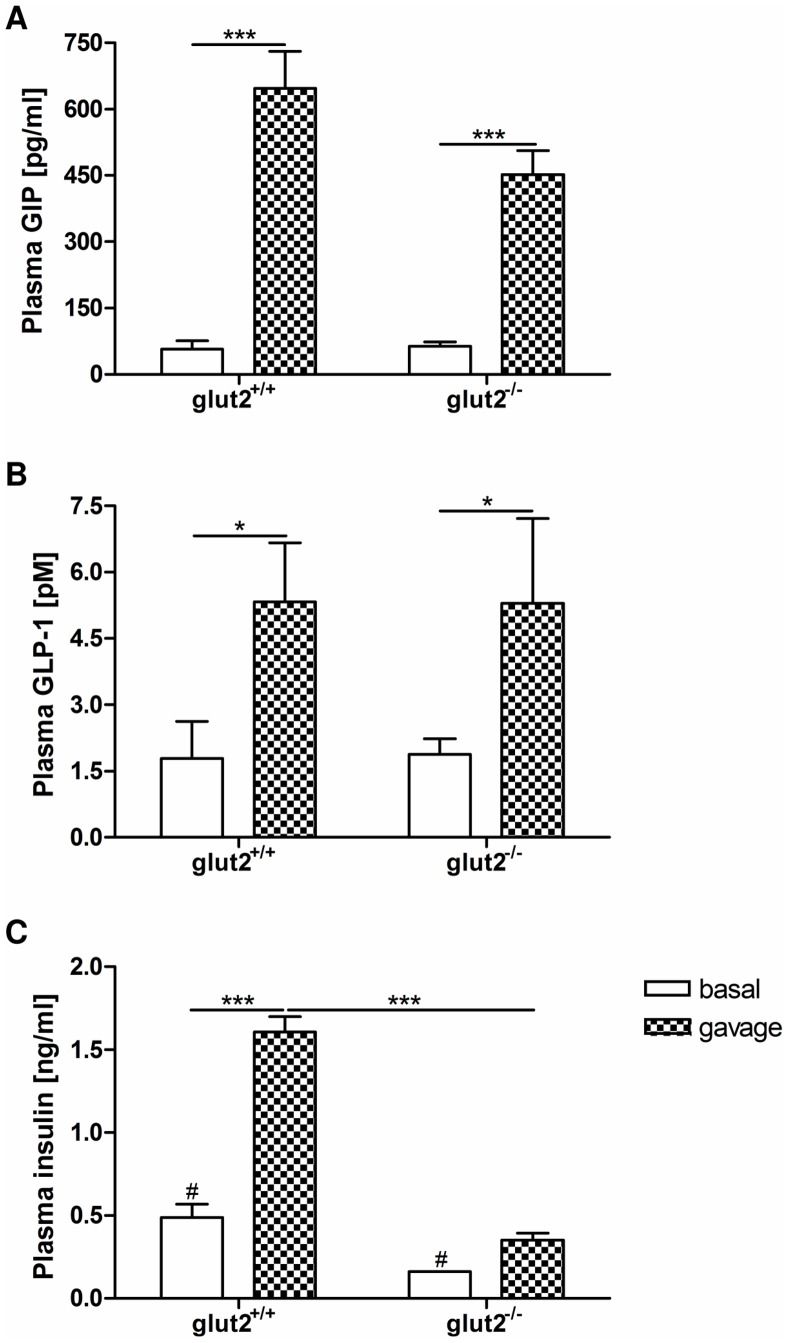
Absence of GLUT2 affects insulin secretion but not GIP or GLP-1 levels after glucose gavage. In *glut2^+/+^* and *glut2^−/−^* mice receiving an oral glucose gavage (4 g/kg) plasma levels of GIP, GLP-1 and insulin were measured before (white bars) and 15 minutes after the bolus (plaid bars). (A) Total GIP concentrations in the fasting state (basal) and after glucose gavage. (B) Active GLP-1 concentrations in the fasting state and after glucose gavage. (C) Insulin concentrations in the fasting state and after glucose gavage. Values are expressed as mean ± SEM. Statistical analyses were performed using 2-way ANOVA with Bonferroni post-test for comparison of GIP and insulin concentrations. ^#^ *p*<0.05, *** *p*<0.001. 2-way ANOVA was used for comparison of GLP-1 levels. * *p*<0.05. N = 3–4 mice per group.

GIP levels in the plasma of *glut2^+/+^* animals were elevated more than 11-fold after the glucose bolus while the increase in *glut2^−/−^* mice was only 7-fold (Fig. 4A). The GLP-1 response to glucose did not differ between wild type (3-fold) and knockout mice (2.8-fold) (Fig. 4B). In mice lacking GLUT2, basal insulin concentrations were reduced by 67% compared to wild type animals. Altered fasting insulin levels in these mice have been reported previously [27]. *Glut2^+/+^* mice challenged with glucose exhibited 3.3-fold higher plasma insulin levels compared to the basal state and at the same time 4.6-fold higher insulin concentrations when compared to animals lacking GLUT2 (Fig. 4C).

### Apical Protein Densities of SGLT1 and GLUT2 Remain Unchanged After Glucose Gavage

Western blot analysis was performed to assess whether SGLT1 and GLUT2 levels in brush border membranes (BBM) change in response to the high glucose load. BBM were isolated from mucosal scrapings obtained from wild type as well as appropriate *sglt1* and *glut2* knockout mice before (basal level) and after the glucose gavage followed by Western blot analysis and determination of protein densities. Enrichment of BBM was ∼10-fold based on specific activity of the apical marker alkaline phosphatase (data not shown). High glucose concentrations did not alter SGLT1 protein abundance in BBM of either *sglt1^+/+^* or *glut2^+/+^* and *glut2^−/−^* mice when compared to basal levels (Table 1 and Fig. S1 A and B).

**Table 1:** Protein density of SGLT1 in brush border membranes of *sglt1* and *glut2* wild type mice and appopriate knockout littermates.

Mouse model		fold change	*p*-value
*sglt1^+/+^*	basal	1.0±0.10	0.18
	gavage	0.9±0.05	
*sglt1^−/−^*	basal	−	−
	gavage	−	
*glut2^+/+^*	basal	1.0±0.14	0.47
	gavage	1.0±0.14	
*glut2^−/−^*	basal	0.8±0.08	0.40
	gavage	0.9±0.17	

BBM were isolated from mucosal scrapings before (basal) and after intragastric glucose gavage (gavage). Samples were stained for SGLT1 and expression levels were quantified by densitometry. Values representing fold changes are expressed as mean ± SEM. Statistical analyses were performed using one-tailed unpaired t-test to compare SGLT1 protein abundance before and after glucose gavage in wild type and knockout mice, respectively. N = 3 mice per group.

Although we detected GLUT2 in the apical membrane fraction we assume that it was almost exclusively derived from a basolateral contamination as we also detected considerable quantities of the basolateral marker Na-K-ATPase in all BBM preparations (Fig. S1 C and D). The proportion of this carry-over of basolateral membrane was comparable in all experiments performed. However, apical GLUT2 protein levels remained unchanged after glucose gavage in both, *glut2^+/+^* and *sglt1^+/+^* as well as in *sglt1^−/−^* mice when compared to basal levels (Table 2 and Fig. S1 C and D). Thus, high luminal glucose concentrations provided by gavage did not influence either SGLT1 or GLUT2 protein abundance in brush border membrane fractions.

**Table 2:** Protein density of GLUT2 in brush border membranes of *sglt1* and *glut2* wild type mice and appropriate knockout littermates.

Mouse model		fold change	*p*-value
*sglt1^+/+^*	basal	1.0±0.27	0.13^a^
	gavage	0.6±0.02	
*sglt1^−/−^*	basal	0.5±0.13	0.24
	gavage	0.4±0.02	
*glut2^+/+^*	basal	1.0±0.24	0.47
	gavage	0.7±0.09	
*glut2^−/−^*	basal	−	−
	gavage	−	

BBM were isolated from mucosal scrapings before (basal) and after mice received the glucose gavage (gavage). Samples were stained for GLUT2 and protein levels were quantified by densitometry. Values representing fold changes are expressed as mean ± SEM. Statistical analyses were performed using one-tailed unpaired t-test ± Welch’s correction^a^ for comparison of GLUT2 protein expression before and after glucose gavage in wild type and knockout mice, respectively. N = 3 mice per group.

### Intestinal Immuno-staining Confirms Apical SGLT1 and Basolateral GLUT2 Localization

**Figure 5:**
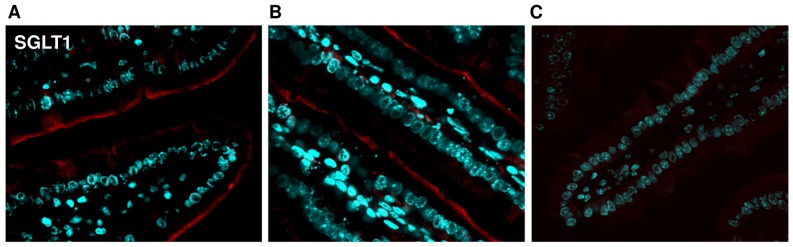
SGLT1 is located in the apical membrane. Jejunal samples from *sglt1^+/+^* mice before and after glucose gavage, respectively, as well as from and *sglt1^−/−^* littermates were stained for SGLT1 (red). Nuclei were stained with DAPI (blue). Apical localization of SGLT1 in *sglt1^+/+^* mice (A) before and (B) after glucose challenge and in (C) *sglt1^−/−^* littermates.

**Figure 6:**
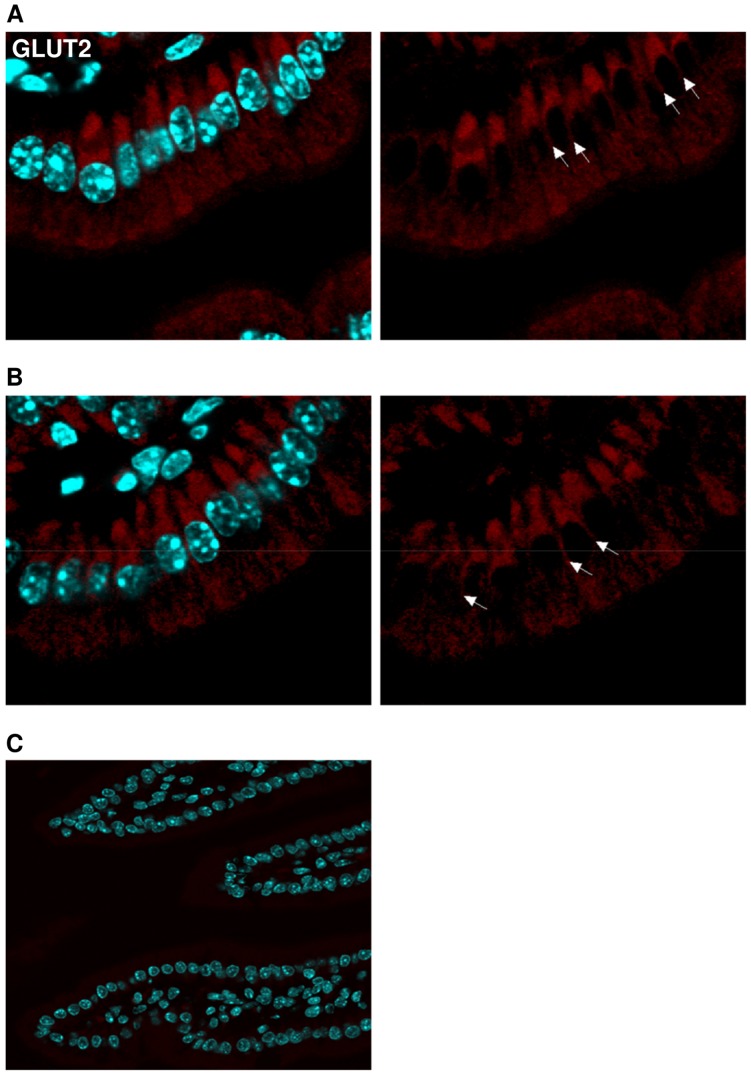
GLUT2 is not located in the apical but basolateral membrane. Jejunal samples from *glut2^+/+^* animals before and after glucose gavage, respectively, as well as from *glut2^−/−^* littermates were stained for GLUT2 (red). Nuclei were stained with DAPI (blue). Basolateral localization (arrows) of GLUT2 in *glut2^+/+^* mice (A) in the basal state and (B) after glucose administration. (C) GLUT2 staining is absent in *glut2^−/−^* littermates.

Jejunal sections from *sglt1* and *glut2* wild type and appropriate knockout mice before and after challenge with 4 g/kg glucose were stained for SGLT1 and GLUT2 to determine the localization of SGLT1 and GLUT2 in enterocytes. As suggested by Western blots, SGLT1 was exclusively located in the apical membrane of *sglt1^+/+^* mice with no change before (basal) and after glucose gavage (Fig. 5A and B). In *sglt1^−/−^* littermates, SGLT1 staining was absent (Fig. 5C). The GLUT2 protein was not detected in the apical but in the basolateral membrane in *glut2^+/+^* mice that was also not changed by glucose gavage (Fig. 6A and B), thereby substantiating the notion that GLUT2 immuno-reactivity in BBM detected in Western blots originated from basolateral cross-contamination. The specificity of the antibody was demonstrated by lack of GLUT2 staining in *glut2^−/−^* littermates (Fig. 6C).

## Discussion

Whereas Wright and colleagues attribute intestinal glucose absorption solely to SGLT1 [2], Kellett and co-workers proposed that GLUT2 is recruited into the apical membrane to mediate bulk absorption of glucose at higher luminal glucose concentrations [18]. More recently, Gorboulev *et al.* concluded from studies in mice that only a minor fraction of overall glucose absorption can be assigned to apical GLUT2 [5]. Within the studies described here we did not find any evidence for apical GLUT2 under our dietary and experimental conditions. Although we cannot completely exclude that a small amount of GLUT2 is present in the brush border membrane its quantity did not change following a glucose load. As opposed to studies in rats and mice using intestinal perfusion [12]–[17], [28] or *in vitro* methods such as everted gut rings [20] we used a glucose gavage including glucose radiotracer to assess intestinal glucose transport processes *in vivo* in mice. The roles of SGLT1 and GLUT2 in intestinal glucose transport and incretin hormone secretion have been assessed before by different groups including the use of either SGLT1- [5] or GLUT2-deficient animals [20], [24] or by applying the respective inhibitors, i.e. phlorizin [23] or phloretin [25]. In the present studies we used SGLT1 and GLUT2 knockout models for a direct comparison with animals fed identical diets and analyses performed under identical experimental conditions.

Our studies in SGLT1-deficient mice revealed that glucose absorption as determined by radiotracer contents in intestinal tissues was significantly reduced as were plasma tracer amounts and consequently the rise in blood glucose after glucose gavage. These data confirm the prime role of SGLT1 in intestinal glucose uptake even at high glucose concentrations and are further substantiated by abolition of *α*-methyl-D-glucopyranoside uptake into everted gut rings prepared from *sglt1* knockout mice [5]. Despite the marked reduction in glucose uptake in SGLT1-deficient mice there was still some 20% residual glucose tracer detected in intestinal tissues and blood. Furthermore, we observed a modest glucose-induced increase in blood glucose levels in *sglt1* knockout animals. This resulted obviously from the residual glucose uptake as indicated by radiolabeled tracer appearance in plasma but also from stress-induced glucose release from the liver as described before [5]. Although we cannot clarify how glucose entered the tissue and subsequently circulation it is unlikely that this is mediated by apical GLUT2 since its recruitment into the apical membrane was shown to depend on SGLT1 [29].

Recent studies have convincingly demonstrated that SGLT1 is also involved in glucose-induced secretion of incretin hormones [5], [23], [30], [31]. We here confirm that GIP and GLP-1 responses in the absence of SGLT1 are almost completely abolished following intragastric glucose administration. These findings clearly endorse the pivotal role of SGLT1 as a sensor for incretin secretion [5]. Moreover, the almost complete loss of GIP and GLP-1 responses in *sglt1* knockout animals argues against a significant role of other glucose transporters such as GLUT2 [25] to function as a glucose sensor. Consistent with findings reported by Gorboulev *et al.* [5] there was a significant decrease in the rise of insulin levels after glucose gavage in SGLT1-deficient mice. This may be attributed to the lack of incretin response and/or the diminished rise in blood glucose levels.

In accordance with its dominant basolateral localization, loss of GLUT2 resulted in the present studies in an impaired glucose efflux from intestinal cells into circulation. This was demonstrated by accumulation of radiolabeled glucose in intestinal tissues of *glut2* knockout animals after gavage which in turn resulted in reduced tracer contents in the plasma and thus a lower increase in blood glucose levels. In contrast, unaltered blood glucose levels in GLUT2-deficient mice after an intraperitoneal injection of 1 g/kg glucose were described by Thorens *et al.* [27]. Stümpel *et al.* reported an unaltered appearance of glucose in portal vein blood in the same knockout model in which glucose absorption was determined as the appearance of glucose in portal blood using an intestinal perfusion. Based on the finding of unaltered glucose appearance it was concluded that a pathway involving the endoplasmic reticulum and glucose-6-phosphatase controlled glucose efflux from enterocytes but not GLUT2 [32]. Our studies using the intragastric gavage however support the “classical” role of GLUT2 as an efflux system as indicated in physiology text books.

GLUT2 was also proposed to function as a sensor linking luminal glucose to hormone secretion. Mace and co-workers performed intestinal perfusion experiments in rats including the use of GLUT2 inhibitors which led to markedly reduced GIP and GLP-1 levels in serosal fluid samples [25]. This, however, is contradictory to the findings by Cani *et al.* demonstrating an involvement of GLUT2 in GLP-1 but not GIP secretion in mice [24]. In our studies, the rise in plasma GIP concentrations following the glucose gavage in *glut2* knockout mice accounted for 70% of that in wild type mice whereas the increase in GLP-1 levels was independent of genotype. The reason for this discrepancy might be the duration of fasting (overnight *vs.* 6 hours) as well as the amount (3 g/kg *vs.* 4 g/kg) and way of glucose administration (stomach catheter *vs.* feeding tube). Furthermore, we quantified active GLP-1 in peripheral blood samples whereas Cani *et al.* measured total GLP-1 in hepatoportal vein plasma [24]. However, hepatoportal GLP-1 levels are not necessarily reflected and detected in systemic blood [33]. Moreover, it has not been demonstrated yet that GLUT2 resides in apical membranes of K- or L-cells in which it could function as a sensor for luminal glucose. Our data obtained in mice lacking GLUT2 suggest that this protein has only a minor – if at all any – role in glucose-induced incretin secretion. Animals lacking GLUT2 displayed lower basal insulin concentrations as previously reported by Thorens *et al.* and a significantly reduced increase in blood glucose despite the fact that they were described as to have normal glucose tolerance and insulin secretory response during hyperglycemic clamps [27]. Our findings are similar to those of Cani *et al.* showing markedly diminished plasma insulin levels in GLUT2-deficient mice [24]. It is likely that the reduced systemic influx of glucose from the intestine leads via the reduced blood glucose levels to the diminished insulin secretion.

Isolated BBM were used to assess apical SGLT1 and GLUT2 protein abundances before and after the glucose gavage. Despite the high glucose load, protein densities remained unchanged. The finding contradicts the increase of SGLT1 protein levels reported by Gorboulev *et al.* [5]. This discrepancy may originate from the amount of glucose administered (4 g/kg *vs.* 6 g/kg) and/or the duration of fasting prior to the gavage. SGLT1 protein levels and the response to glucose may differ when animals are fasting for 6 hours as in the present study or for 18 hours as used by Gorboulev *et al.* [5]. Furthermore, the time of day when the gavage is administered might play a role since SGLT1 shows diurnal rhythmicity [34]. It is possible that in the present study basal SGLT1 expression levels were higher due to shorter duration of fasting and the later time of the gavage (2 p.m. *vs.* 11 a.m.).

For GLUT2, we detected some protein in BBM but this resulted mainly from cross-contamination with basolateral membranes as demonstrated by considerable amounts of Na-K-ATPase (Fig. S1C and D) and e-cadherin (data not shown). All attempts to further enrich the BBM fraction and/or to reduce the basolateral membrane contamination failed or brought protein levels below detection limits. We thus cannot exclude that some of the GLUT2 protein detected is indeed in the apical membrane although immunofluorescence staining revealed GLUT2 to be located in basolateral membranes. However, if GLUT2 would indeed reside in apical membranes an increase in protein density after the glucose gavage was expected according to model proposed [18], [20] and this increase should not be found in animals deficient of SGLT1 [29]. The lack of both, the increase in GLUT2-density in BBM after the gavage and the lack of a difference between *sglt1* knockout and wild type mice thus argues strongly against a role of apical GLUT2 in overall glucose absorption in mice – at least under the experimental conditions applied here. We would like to stress that our conditions with a 40% glucose solution given by gavage resulted in a luminal glucose concentration of approximately 160 mM (see Methods) that should have been more than sufficient to evoke GLUT2 trafficking [18], [20], [28], [35].

In conclusion, we confirm the prime role of SGLT1 in intestinal glucose absorption in mice challenged with a high intragastric glucose load. We also support with our findings that GLUT2 is involved in basolateral glucose efflux but we could not find evidence for any role of apical GLUT2. Moreover, glucose-mediated incretin hormone secretion was shown to depend on SGLT1 with only a minor contribution of GLUT2. We would like to stress that all animals in the present studies were fed a sugar-free diet and this may also be important for the results obtained. For example, Gouyon *et al.* [20] failed to detect apical GLUT2 in chow-fed animals but demonstrated GLUT2 trafficking when mice were fed a fructose-rich diet for 5 days. The controversial findings reported for GLUT2 in intestinal glucose uptake might thus result from species differences, diet effects and examination methods. In this respect one can assume that the controversy of whether GLUT2 is recruited into the apical membrane as the “phlorizin-insensitive” component may continue.

## Supporting Information

Figure S1 SGLT1 and GLUT2 protein abundance in brush border membranes are unaffected by glucose gavage. Samples for Western blot were obtained from *sglt1* and *glut2* wild type and respective knockout mice. BBM were isolated from mucosal scrapings before (basal, white bars) and after the 4 g/kg glucose bolus (glucose, plaid bars). Samples were stained for SGLT1 and GLUT2 and quantified by densitometry. SGLT1 abundance in (A) *sglt1^+/+^* and *sglt1^−/−^* as well as in (B) *glut2^+/+^* and *glut2^−/−^* animals. Expression of GLUT2 in (C) *sglt1^+/+^* and *sglt1^−/−^* as well as in (D) *glut2^+/+^* and *glut2^−/−^* mice. Values are expressed as mean ± SEM. Statistical analyses were performed using unpaired t-test to compare protein abundance of SGLT1 and GLUT2 before and after gavage in wild type and knockout animals, respectively. N = 3 mice per group.
(TIFF)
