# Supplementary figures and images for: The Role of SGLT1 and GLUT2 in Intestinal Glucose Transport and Sensing
Source: PLoS One. 2014 Feb 26;9(2):e89977. doi: 10.1371/journal.pone.0089977 (PMC3935955; doi:10.1371/journal.pone.0089977)

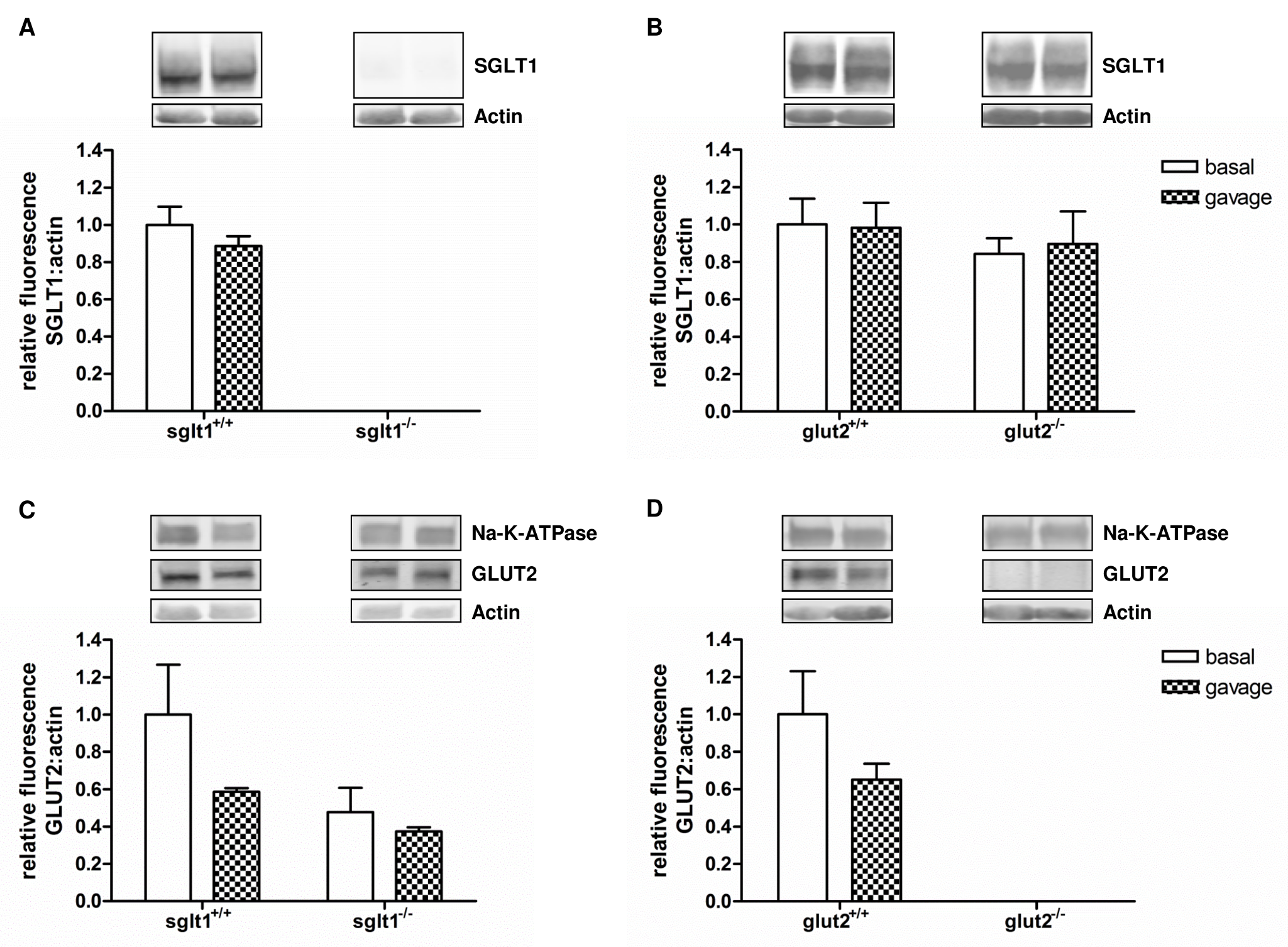

Supplement: Figure S1 — SGLT1 and GLUT2 protein abundance in brush border membranes are unaffected by glucose gavage. Samples for Western blot were obtained from sglt1 and glut2 wild type and respective knockout mice. BBM were isolated from mucosal scrapings before (basal, white bars) and after the 4 g/kg glucose bolus (glucose, plaid bars). Samples were stained for SGLT1 and GLUT2 and quantified by densitometry. SGLT1 abundance in (A) sglt1+/+ and sglt1−/− as well as in (B) glut2+/+ and glut2−/− animals. Expression of GLUT2 in (C) sglt1+/+ and sglt1−/− as well as in (D) glut2+/+ and glut2−/− mice. Values are expressed as mean ± SEM. Statistical analyses were performed using unpaired t-test to compare protein abundance of SGLT1 and GLUT2 before and after gavage in wild type and knockout animals, respectively. N = 3 mice per group. (TIFF) [file pone.0089977.s001.tiff]
